# Proposal for coordinated health research in PFAS-contaminated communities in the United States

**DOI:** 10.1186/s12940-017-0321-6

**Published:** 2017-11-14

**Authors:** Thomas A. Bruton, Arlene Blum

**Affiliations:** 1 Green Science Policy Institute, Berkeley, CA USA; 2 Department of Chemistry, University of California at Berkeley, Berkeley, CA USA

**Keywords:** PFAS, Highly fluorinated chemicals, PFOA, PFOS, Drinking water, Health study, Biomonitoring

## Abstract

The drinking water of more than six million Americans in numerous communities has been found to contain highly fluorinated chemicals at concentrations of concern. Certain of these chemicals, including perfluorooctanoic acid and perfluorooctane sulfonic acid, are known to be persistent, bioaccumulative, and associated with adverse health outcomes in humans and animal models. The possible health impacts of exposure to highly fluorinated chemicals are of great concern to communities whose water has been impacted. Community members want information, and are asking for biomonitoring, exposure pathway analysis, and health studies. Governmental agencies are striving to deal with these multiple concerns in the face of information and resource constraints. We propose the development of a high-level research strategy to maximize what can be learned about health effects of highly fluorinated chemicals and methods to reduce or eliminate exposure. We suggest coordinating the research across multiple communities for greater statistical power. If implemented, such a strategy could help to generate information and evidence integration to enable regulatory decision making and contribute to reducing future exposures.

## Background

We write as scientists, public health officials, and physicians (see Appendix 1 for full list of signatories) to propose a coordinated plan of action for the study of U.S. communities whose drinking water is contaminated with highly fluorinated chemicals (per- and polyfluoroalkyl substances, PFAS; sometimes known as PFCs). This highly persistent and potentially toxic class of chemicals, including perfluorooctanoic acid (PFOA), perfluorooctane sulfonic acid (PFOS), and many related substances, have been released into the environment worldwide, resulting in widespread contamination of drinking water supplies. For example, more than six million Americans in numerous communities have been exposed to these chemicals at concentrations of concern. A research agenda that is coordinated across the many impacted communities in the U.S. is needed to maximize what can be learned from these unfortunate exposures. First, the impacted communities urgently need clean drinking water. As they receive it, the levels of PFAS contaminants in people’s bodies should decrease. Second, we need to learn from the current unfortunate situation to understand the implications of these exposures and their potential link to health impacts. A coordinated program of exposure analysis, biomonitoring, health studies, and medical monitoring should help regulators set appropriate health advisory levels and contribute to preventing similar future contamination, both in the U.S. and internationally.

## Extent of contamination

The extent of PFAS contamination became known after PFOA, PFOS, and others were detected at relatively high concentrations in public drinking water systems around the country through a survey led by the U.S. Environmental Protection Agency (EPA) between 2013 and 2015 [1]. In May 2016, after more than a decade of study on exposure and effects of PFAS, EPA issued a lifetime health advisory level of 70 parts per trillion for the sum of PFOS and PFOA, the two PFASs detected at the highest levels in humans and the environment [2]. The drinking water of millions of Americans was found to contain these chemicals at concentrations above this safety level [1]. Major sources include production, use, and disposal of PFAS at manufacturing sites, as well as military fire training areas, civilian airports, and wastewater treatment plants. Sixty-three communities whose water had been tested and found to contain high levels were informed by the EPA of the potential for harm [3]. Biomonitoring has shown that people living in areas with contaminated drinking water are more likely to have elevated concentrations in their blood [4–7].

## Potential for harm

The possible adverse health impacts are of great concern to members of affected communities. In human observational (epidemiological) studies, PFOA and/or PFOS have been associated with kidney and testicular cancer, decreased birth weight, thyroid disease, decreased sperm quality, high cholesterol, pregnancy-induced hypertension, asthma, ulcerative colitis, and decreased response to vaccination [5]. In animal studies, one or both of these chemicals cause liver toxicity, thyroid toxicity, testicular, pancreatic, thyroid, and liver tumors, obesity, immune suppression, and reproductive and developmental toxicity including altered mammary gland development, reduced ossification, accelerated puberty, resorption of developing fetus, and mortality and delayed development of offspring [5]. Exposure to these chemicals is especially harmful during critical windows of fetal development. PFOA, PFOS, and other long-chain (from 6 to 12 fluorinated carbon atoms) PFAS are of particular concern because they remain in the body for many years after exposure ends [8]. Production of long-chain PFAS has been phased out in the United States, Western Europe, and Japan, and the use of these legacy chemicals is being replaced by short-chain perfluoroalkyl acids (3–5 fluorinated carbon atoms) and other subclasses of PFAS, such as perfluoroalkyl ether carboxylic acids and sulfonic acids. These replacement compounds have been detected in soil and groundwater at high levels and in drinking water supplies [1], but have not been studied for their health effects in humans.

## Current situation

State health departments and the federal Agency for Toxic Substances and Disease Registry (ATSDR), with limited information and resources, are striving to deal with multiple concerns arising from the contamination of the environment, humans, livestock, and wildlife. Affected communities want clean drinking water, and information about their exposures and potential long-term health effects. Community members, who have been using contaminated water for drinking, food preparation, showering, washing, and watering gardens, sometimes for decades, want to know their blood-levels, what those measurements mean for their family (especially their children), and how to reduce them. Communities are asking for biomonitoring, exposure pathway analysis, and health studies. At present, studies are being initiated in a few locations to address the needs of the community or interests of the researcher. However, the scope and objectives of these studies vary. Coordination between the many different community efforts, academic studies, and public health investigations is needed to address the interests and needs of all stakeholders.

## A coordinated research agenda for health studies in PFAS-contaminated communities in the U.S.

Much of the power of epidemiology studies and exposure science depends on the number of participants: the larger the number of participants, the greater the potential for useful results. The availability and timing of both exposure and health outcome measurements are key factors that affect the utility of data. If biomonitoring, exposure analysis, health recording, and epidemiological research were to be coordinated across multiple impacted communities, a wealth of useful information on health effects and methods to reduce or eliminate exposure from PFAS could be obtained. Questions include:

- Which legacy and current-use PFAS have communities been exposed to, and what were the levels and durations of these exposures?
- What health effects may be associated with these exposures?
- What levels of exposure might trigger such health effects?
- Which life stages are most sensitive to the effects of PFAS exposure?
- What is the relationship between the concentrations in drinking water and in humans?
- How does the drinking water exposure route compare to other routes such as ingestion of food or dust and dermal absorption in terms of magnitude and potential health effects?
- What are the rates at which the chemicals are cleared from the human body?
- What, if anything, can be done to mitigate exposure and the probability of adverse health effects, especially in children?

## Precedent: C8 study and what was learned

One model for coordinated studies of large populations exposed to PFAS is the C8 Science Panel. Beginning in 2006, nearly 70,000 community members in the Ohio River Valley whose drinking water had been contaminated by PFOA for decades were studied retrospectively by the C8 Panel [7]. This independent panel of three leading epidemiologists collaborated with local medical professionals and many other researchers to design a series of complementary studies to assess the probable links between PFOA and disease. Their results, which found probable links between PFOA exposure and six human diseases, as well as associations with several other health endpoints, substantially contributed to what is known about the health effects of this chemical [9].

As an adjunct to the above, a complementary approach would be to bring together common data from existing studies as does the NIH ECHO program for children’s health.

**We propose the following:**

- Invite experts in fields including epidemiology, medical ethics, biostatistics, immunology, toxicology, exposure science, cancer, and endocrinology to develop a high-level coordinated research strategy. This strategy would address the needs of affected communities for education, biomonitoring, health studies, and medical monitoring, and would establish clear protocols for disclosure of research findings. This plan for community-based studies would build on the C8 study methodology as well as other current research protocols. This research could also include data from other ongoing studies of the health effects of chemical exposures using large longitudinal cohorts.
- Develop the above research agenda with active participation from members of affected communities.
- Estimate cost of biomonitoring and health studies following the approach developed above.
- Communicate the approach outlined above to communities and decision makers including local and state health departments, federal agencies such as ATSDR and NIEHS, the military, members of Congress and other stakeholders, especially those from impacted areas, and seek public comment and input.
- Work with the federal government agencies already involved in PFAS research to initiate these coordinated studies.
- A likely mechanism for development of such a high-level research strategy was included in proposed amendments to the 2018 U.S. Defense authorization bill. The intent of these amendments was to authorize a five-year national study of PFOA and PFOS exposures resulting from military use of PFAS-containing firefighting foam. While the passage of these amendments is a step in the right direction, this study, as described, is likely insufficient. If and when the study is carried out, its scope should be expanded to include a wider range of PFASs than PFOA and PFOS. Such a study could also be improved by including all federal agencies with relevant expertise, including the NIEHS.

## Conclusions

PFAS and other environmental contaminants impact nearly all of us. Indeed, highly fluorinated chemicals have been detected in the blood of more than 95% of Americans [10]. Communities worldwide are facing contamination problems similar to those in the U.S. The current widespread contamination of drinking water provides an opportunity to better understand many types of PFAS chemicals, and their human health effects.

We as scientists and public health professionals support the development of a coordinated research strategy to learn as much as possible from the unfortunate exposure of millions of Americans to PFASs in their drinking water. Action is needed now to generate the information and evidence integration to enable regulatory decision making. This coordinated research strategy would also contribute to reducing exposure to PFASs from drinking water and other sources.

## Appendix

### SIGNATORIES

**(Institutional names provided for reference only)**

**Lutz Ahrens**, PhD, Associate Professor,

Department of Aquatic Sciences and Assessment

Swedish University of Agricultural Sciences

Uppsala, Sweden

**Alfredo Alder**, PhD,

Department of Environmental Chemistry

EAWAG

Dübendorf, Switzerland

**David Andrews**, PhD, Senior Scientist

Environmental Working Group

Washington, DC, USA

**Andrea Baccarelli**, M.D. Ph.D., Professor

Columbia University Mailman School of Public Health

New York, New York, USA

**Urs Berger**, PhD, Senior Scientist

Department of Analytical Chemistry

Helmholtz-Centre for Environmental Research - UFZ

Leipzig, Germany

**Joseph M. Braun**, PhD, Assistant Professor Department of Epidemiology

Brown University School of Public Health

Providence, Rhode Island, USA

**Phil Brown**, PhD, University Distinguished Professor

of Sociology and Health Sciences

Northeastern University

Boston, Massachusetts, USA

**Courtney Carignan**, PhD, Assistant Professor

Department of Food Science and Human Nutrition

Department of Pharmacology and Toxicology

Michigan State University

East Lansing, Michigan, USA

**David O. Carpenter**, MD, Director

Institute for Health and the Environment

A Collaborating Centre of the World Health Organization

University at Albany

Rensselaer, New York, USA

**Perry Cohn**, PhD MPH, Research Scientist (Retired)

New Jersey Department of Health

Trenton, New Jersey, USA

**Ian Cousins**, PhD, Professor and Group Leader

Department of Analytical Chemistry and Environmental Sciences

Stockholm University

Stockholm, Sweden

**Robert Delaney**, Department of Defense and State Memorandum of Agreement Coordinator

Michigan Department of Environmental Quality

Michigan, USA

**Jamie DeWitt**, PhD, Associate Professor

Department of Pharmacology and Toxicology

Brody School of Medicine, East Carolina University

Greenville, North Carolina, USA

**Miriam Diamond**, PhD, Professor,

Department of Geography & Planning and

Department of Earth Sciences

University of Toronto

Toronto, Ontario, Canada

**Alan Ducatman**, MD MSc, Professor

School of Public Health,

West Virginia University

Morgantown, WV, USA

**Tracey Easthope**, MPH, Safer Chemicals Leader

Health Care Without Harm

Ann Arbor, Michigan, USA

**Susan Fisher,** PhD, Professor

Department of Obstetrics, Gynecology, and Reproductive Sciences

University of California San Francisco

San Francisco, CA, USA

**Russ Hauser,** MD, ScD, MPH

Acting Chair, Department of Environmental Health

Frederick Lee Hisaw Professor of Reproductive Physiology

Professor of Environmental and Occupational Epidemiology

Harvard T.H. Chan School of Public Health, Harvard University

Cambridge, Massachusetts, USA

**Rakesh Kanda**, PhD, Professor

Department of Life Sciences

Brunel University London

Uxbridge, United Kingdom

**Detlef Knappe**, PhD, Professor

Department of Civil, Construction, and Environmental Engineering

North Carolina State University

Raleigh, North Carolina, USA

**Philip J. Landrigan**, MD, MSc, FAAP

Dean for Global Health

Professor of Environmental Medicine, Public Health and Pediatrics

Arnhold Institute for Global Health, Icahn School of Medicine at Mount Sinai

New York, New York, USA

**Rainer Lohmann**, PhD, Professor

Graduate School of Oceanography

University of Rhode Island,

Kingston, Rhode Island, USA

**Matthew MacLeod**, PhD, Professor

Department of Analytical Chemistry and Environmental Sciences

Stockholm University

Stockholm, Sweden

**Olwenn Martin**, PhD, Post-doctoral Research Fellow

Department of Life Sciences

Brunel University London

Uxbridge, United Kingdom

**Graham Peaslee**, PhD, Professor

Department of Physics

University of Notre Dame

Notre Dame, Indiana, USA

**Laurel Schaider**, PhD, Research Scientist

Silent Spring Institute

Newton, MA, USA

**Martin Scheringer**, DSc, Professor

RECETOX

Masaryk University

Brno, Czech Republic

**Emma Schymanski**, PhD, Principle Investigator

Luxembourg Centre for Systems Biomedicine,

University of Luxembourg

Luxembourg

**Ted Schettler**, MD MPH, Science Director

Science and Environmental Health Network

Bolinas, California, USA

**Margaret Sedlak**, MS, Senior Program Manager

Bay Regional Monitoring Program

San Francisco Estuary Institute

Richmond, California, USA

**Elsie Sunderland**, PhD, Professor

Harvard John A. Paulson School of Engineering and Applied Sciences

Harvard University

Cambridge, Massachusetts, USA

**Rebecca Sutton**, PhD, Senior Scientist

Bay Regional Monitoring Program

San Francisco Estuary Institute

Richmond, California, USA

**Marc-André Verner**, PhD, Assistant Professor

Université de Montréal Public Health Research Institute (IRSPUM)

Department of Occupational and Environmental Health

Université de Montréal

Montreal, Quebec, Canada

**Frank A. von Hippel**, PhD, Professor of Ecotoxicology

Department of Biological Sciences

Northern Arizona University

**Graham White**, PhD, Senior Chemist Evaluator

New Substances Assessment and Control Bureau

Health Canada

Ottawa, Ontario, Canada

**Karin Wiberg**, PhD, Professor in Organic Environmental Chemistry

Department of Aquatic Sciences and Assessment

Swedish University of Agricultural Sciences

Uppsala, Sweden

**Tracey Woodruff**, PhD, MPH

Professor and Director, Program on Reproductive Health and the Environment

Department of Ob/Gyn & the Institute for Health Policy Studies

University of California, San Francisco

## Abbreviations

ATSDR: Agency for toxic substances and disease registry
EPA: U.S. environmental protection agency
NIEHS: National Institute of Environmental Health Sciences
PFAS: Per- and polyfluoroalkyl substances
PFC: Perfluorinated chemical
PFOA: Perfluorooctanoic acid
PFOS: Perfluorooctane sulfonic acid
